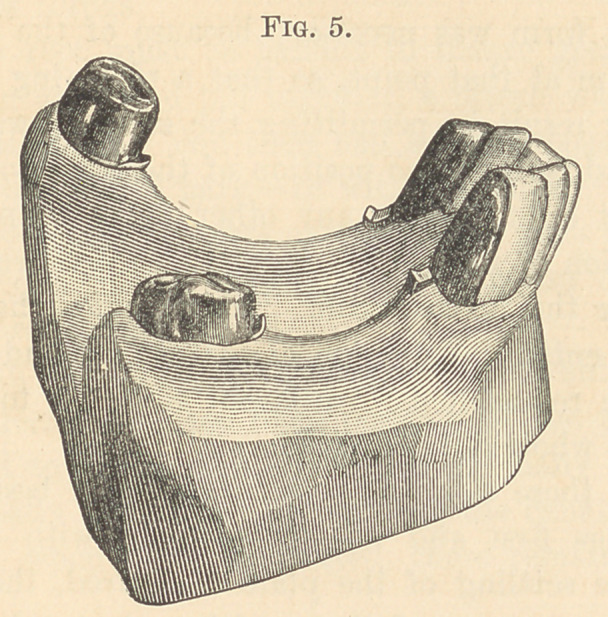# Device for Extension Crown

**Published:** 1904-06

**Authors:** Henry W. Gillett

**Affiliations:** New York


### International Dental Journal.

Vol. XXV. June, 1904. No. 6.

### Original Communications.[1]

### DEVICE FOR EXTENSION CROWN.[2]

BY HENRY W. GILLETT, D.M.D., NEW YORK.

Mr. President and Gentlemen,—I am moved to make these few remarks, which can scarcely be dignified by the name of a paper, by the following train of circumstances.

I received recently, from Chicago, a circular describing the conditions to which a so-called extension crown is applicable as an aid in securely anchoring lower partial plates, and offering a license for the use of the patented process for the sum of fifty dollars.

The documents, which very carefully avoided any description of the device, I mailed to Dr. J. N. Crouse for his information, but the point of particular interest in them for me was the name extension crown, because for some years I have been using a device to which the name might apply. Asking one of our leading Chicago dentists about the device offered in the circular led to

[1] The editor and publishers are not responsible for the views of authors of papers published in this department, nor for any claim to novelty, or otherwise, that may be made by them. No papers will be received for this department that have appeared in any other journal published in the country.

[2] Read before the American Academy of Dental Sciences, Boston, February 3, 1904.

my also asking if the device I use is generally known and used, and if it had ever been described so as to safeguard it from patents. His verdict that it ought to be so described and your committee's request for a paper were coincident in time.

The kernel of the matter I want to put before you is the principle involved in the providing of a fixed point of support for certain classes of partial plates. I think most of us are quite ready to admit that fixed bridge-work is less cleanly than removable pieces, but oftentimes the superior firmness it offers is so essential as to more than offset that defect, and lead to its use, where we would prefer to advise partial plates. Removable bridges, strictly so classed, often call for so much destructive cutting as to rule them out. I think you will bear me out in the statement that many times these two points lead to the use of a plate, which the operator recognizes as less effective as a masticator than the bridge that might be used.

Dr. Bonwill showed us years ago one way of overcoming this difficulty when dealing with spaces having molars and bicuspids on each side. You remember, and without doubt practise, his valuable method of supplying a hook or lug to bear on the occlusal ends of adjoining teeth, and so provide rigid support for small pieces, and you are also familiar with Dr. Head's use of it in connection with porcelain work. I recently saw a most successful application of the principle from Dr. Rhein's hands, the large rectangular lug in this case being let into the surface of a large gold filling in a molar, so as to stand flush with the occlusal surface, and its generous proportions and parallel sides serving to steady the piece against torsional strain.

Modifications of the Bonwill lug are to me invaluable, in a certain type of fixed bridge—but that is another story, and one which Dr. S. S. Stowell has told in detail. Minor objections to Dr. Bonwill's way of applying the lug sometimes present in cases of close bite, where convenient space for it cannot be arranged, or where the shape of occlusal surfaces and the angle at which the teeth stand is unsatisfactory.

The place where we need its help most, however, and where it entirely fails us, is in the cases where we want and often must have rigid support if we are to attain even mediocre success in restoring the masticatory apparatus to real usefulness,—namely, those cases where we have only the six front teeth for our anterior support, or often in the lower jaw for the sole support, and where the occlusion is such as to readily allow these teeth to yield and move forward if pressure is made against them. Even in the cases where the Bonwill lug is readily feasible, it seems to me a better application of the principle to place the point of support nearer the neck of the tooth—not that I discard entirely the Bonwill device, for it is often very useful. Placing the point of support at the neck of the tooth often results in a cleaner piece, because it presents less crevices and corners for débris lodgement. It renders possible the balancing of the strains thrown on the supporting tooth, and when the tooth utilized is one of the six anterior teeth it seems the only practicable point for such attachment.

My first use of the device grew out of my need for solving the problem presented by a case in which an upper partial rubber plate carrying molars, bicuspids, and one cuspid, and bearing heavy stress, kept presenting for repairs. It was observed that the plate was constantly driving up and the upper front teeth, by a process familiar to all of you, were being forced forward quite rapidly. A molar on each side at the back gave opportunity for using Bonwill lugs, but until the left lateral (and later the right cuspid) broke off and needed crowning, no stable support could be found at the front. When the lateral crown was made, a modified Richmond provided the needed help, and later, with the same device on the cuspid, stability was gained which revolutionized the conditions in the mouth.

You have, of course, already grasped the point that this support was provided by a projection and strengthening of the top of the gold cap on the palatal side of the crowns, and the insertion in the rubber plate of a bit of gold plate to rest on this projection. Since that I do not remember to have used the principle in connection with rubber plates, but repeatedly I have found it so indispensable in partial gold plate work, that I have extended its application to all cases where it is feasible to provide the support.

The advent of the so-called staple or Marshall crown has been very helpful in providing means for this support, and I do not hesitate to utilize a half-jacket of the staple class on the palatal surface of cuspids, or even centrals if it is needed.

These half-crowns or jackets provide attachment for simple projections, hooks, or hooked arms as needed, upon which the gold plate may rest. The stress is applied in the line in which the tooth is best able to bear stress, and without torsional or wrenching strain, as when a fixed bridge is used. What lateral strain there is can be easily balanced by contact with the artificial appliance near the occlusal surfaces. The teeth utilized for such support are still left in the best condition to withstand the extra strain put upon them.

In Fig. 1 I present at *a* the simple projection from the side of a molar gold crown; at *b*, a hook sometimes useful when there

Fig. 1.

is a possible spreading tendency of the appliance as it rests between the teeth on each side; at *c*, a more extended hook which I have had occasion to use but once; and at *d*, a projection from the band of a gold-banded porcelain crown.

Fig. 2.

Fig. 3.

In Fig. 2 we have the left side, and in Fig. 3 the right side, of the upper jaw of a heavy strong man, for whom it was necessary to shoe with 18-carat gold the upper six front teeth and open the bite. This, of course, called for rigid support against stress tending to drive the appliances towards the gum, and on the right side I did not dare put so long a span on a fixed bridge, and so expose the only remaining molar on that side to such severe stress. Simple lugs like *a*, of Fig. 1, on the molar gold crowns (*f*) and on staple half-crowns (*e*) on the cuspids provided the desired rigid support for the small plates used. On the lower jaw the same principle of support was supplied, using the first bicuspids to support half-jackets, carrying the necessary projections. Lower bicuspids lend themselves readily to such a device, and in patients of the usual age needing them the pulps have generally become small enough to permit of the comparatively small amount of grinding needed on the lingual, mesial, distal, and occlusal surfaces for the proper fitting of staple crowns. In these teeth I often prefer to get my grip on the tooth, with two 21-guage iridio-platinum pins extending into dowel-holes in the occlusal end, rather than to cut the grooves in the sides for the so-called staple.

Fig. 4.

Fig. 5.

Figs. 4 and 5 show a case over which I have had one of the hardest struggles of my professional life, and in which I have gained what I consider a conspicuous degree of success. Previous plates before my first attempt had been worn mostly in the bureau drawer. The first one I made, with elaborate attention to perfection of detail, shared a similar fate. It had Bonwill lugs on the molars, and rested with broad contact against the slanting lingual surfaces of the six unusually thick rooted lower front teeth. The first day, or sometimes two days, in the mouth it would work perfectly, and then it would have to go on furlough till the bruised gum got well. Repeated relieving of the bearing at the sore points did no good. The ridge is large and firm, and because of its firmness bruised easily. Note the square effect of the cutting edges,[1] and you will realize that I had an edge-to-edge incisor bite to deal with, and the result of a day's mastication on the plate was to force the front teeth outward just a little, and that little allowed impingement of the plate upon the gum sufficient to cause trouble.

The problem was solved in this way. A gold crown was made for the left molar, and it and the gold crown already on the right one were provided with hooks like *b* of Fig. 1. The lower edge of the clasps (*g*) engaged these hooks so that they helped to resist any tendency of the plate to move forward. The lingual and approximal sides of the cuspids were provided with gold jackets of "staple" crowns, anchored by grooves cut into the sides of the teeth. The gold was cut away till it did not show at all on the labial aspect and at the lower edge a hooked arm like *c* of Fig. 1 was soldered. This form was necessary because of the sharp upward slant of the gum at that point, so that a plain lug on that angle would probably result in permitting the same forward movement of the teeth, and because the position of the teeth was such that it was found that a lug set at any other feasible angle interfered with the insertion of the plate. A suitable recess was made in the plate to engage the hooked arm. These devices have solved the problem to the entire satisfaction of the patient and operator, and while the much remodelled plate is not as pretty to look at as at first, it does its whole duty three times a day.

In making these devices, I usually find it best to make all crowns and lugs first and take impressions with the crowns in place. If some settling of the plate is desired, the cast may be run without the crowns and the resulting plaster duplicate of the

---

[1] Not sufficiently pronounced in the cut.

crown may be built up at the point of support as much as is desired, and the plate struck over it.

In such a case as the lower plate just described, it is better to work directly to the crowns themselves, striking up the plate free from contact with the crowns and taking impression with it and the crowns in place, and make the needed additions when you have cast with all parts in place on it. To do this calls for the same absolute accuracy of detail as the highest grade of bridge-work.

## Figures and Tables

**Fig. 1. f1:**
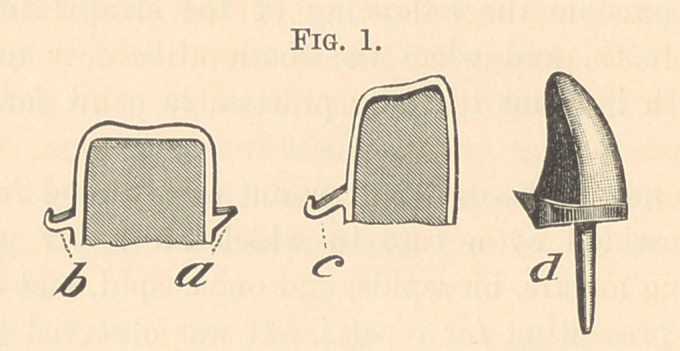


**Fig. 2. f2:**
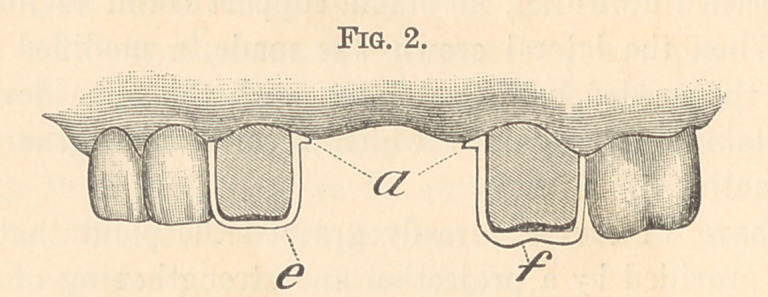


**Fig. 3. f3:**
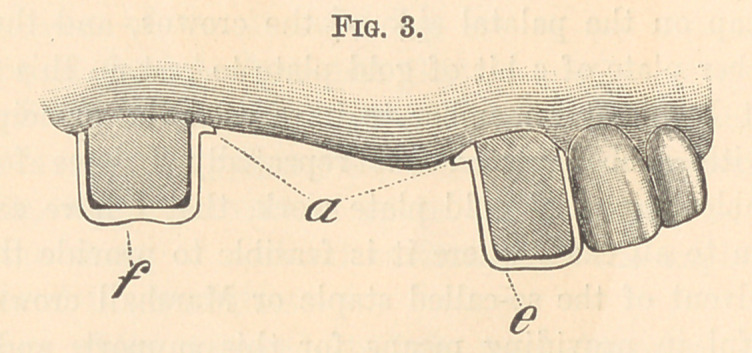


**Fig. 4. f4:**
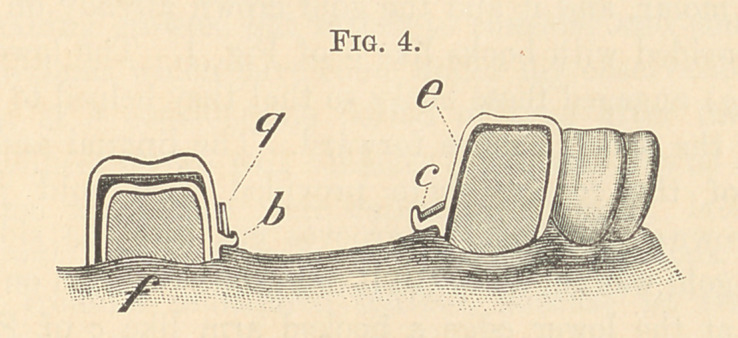


**Fig. 5. f5:**